# Preoperative Nonopioid Analgesia Reduces Postoperative Opioid Consumption After Arthroscopic Surgery: A Systematic Review and Meta-analysis of Randomized Controlled Trials

**DOI:** 10.1177/03635465251396164

**Published:** 2026-01-13

**Authors:** Joshua Dworsky-Fried, Ryley Fowler, Prushoth Vivekanantha, Dan Cohen, Nicole Simunovic, Darren de SA, Olufemi R. Ayeni

**Affiliations:** † Division of Orthopaedic Surgery, Department of Surgery, Western University, London, Ontario, Canada; ‡ Michael G. deGroote School of Medicine, McMaster University, Hamilton, Ontario, Canada; § Division of Orthopedic Surgery, Department of Surgery, McMaster University, Hamilton, Ontario, Canada; Investigation performed at McMaster University, Hamilton, Ontario, Canada

**Keywords:** preoperative, preemptive, analgesia, opioid, pain, arthroscopy, surgery

## Abstract

**Background:**

Orthopaedic surgeons frequently overprescribe opioids after arthroscopic surgery. Previous research has shown reductions in postoperative opioid consumption and pain scores with multimodal nonopioid analgesics. However, the clinical effect of preoperative analgesic strategies has not been fully uncovered.

**Purpose:**

To assess the outcomes of arthroscopic surgery associated with preoperative treatment with nonopioid medications.

**Study Design:**

Systematic review and meta-analysis; Level of evidence, 1.

**Methods:**

Three online databases (PubMed, MEDLINE, Embase) were searched on December 12, 2024, to identify randomized controlled trials investigating the effect of preoperative intervention on pain management following arthroscopic surgery. Extracted data included patient demographics, surgery type, analgesic protocols (ie, type, dosing, timing), adverse effects, postoperative opioid consumption, and pain scores. Postoperative opioid consumption was standardized and converted to oral morphine equivalents. Pain scores were assessed using the visual analog scale (VAS). Weighted means and meta-analyses were conducted to compare postoperative outcomes. Subgroup analyses were performed by analgesic class (COX-2 inhibitors, gabapentin) and surgery type. The quality of studies was assessed with the Risk of Bias 2 tool.

**Results:**

A total of 22 studies were included in this review. The overall pooled mean reduction in postoperative opioid consumption with any preoperative medication type was 4.3 mg of oral morphine equivalents (95% CI, –6.1 to –2.5; *P* < .001; *I*^2^ = 96%) at 24 hours. The pooled mean reduction associated with preoperative COX-2 administration and gabapentin was 4.2 mg (95% CI, –7.9 to –0.5; *P* = .03; *I*^2^ = 93%) and 6.3 mg (95% CI, –9.6 to –3.0; *P* < .001; *I*^2^ = 90%) at 24 hours, respectively. Preoperative COX-2 inhibitors also yielded a statistically significant VAS pain reduction of 0.3 cm (95% CI, –0.5 to –0.02; *P* = .04). Patients undergoing anterior cruciate ligament reconstruction had higher postoperative opioid consumption as compared with general knee or shoulder arthroscopies.

**Conclusion:**

This systematic review demonstrated that preoperative treatment with nonopioid medications, particularly COX-2 inhibitors and gabapentin, is associated with statistically significant reductions in postoperative opioid consumption after arthroscopic surgery. Despite statistically significant findings, observed reductions in postoperative opioid consumption and VAS pain scores may not represent clinically meaningful improvements. The current available literature is highly heterogeneous, indicating the need for high-quality prospective studies to more accurately assess optimal approaches to pain management.

The global opioid crisis continues to escalate, and rates of opioid-related deaths and dependency are on the rise. Prescription opioids remain a substantial contributor, particularly in high-income countries.^14^ In North America, they are implicated in 25% to 33% of opioid-related overdose deaths.^14^ One study found that 32.5% of individuals who died from opioid-related causes between 2013 and 2016 had an active opioid prescription at the time of death.^23^ Orthopaedic procedures are a significant source of prescription opioid exposure.^19^ Although orthopaedic surgeons represent only 2.2% of US physicians, they account for 7.7% of all opioid prescriptions, primarily for postoperative pain management.^4,6^ This underscores the need for standardized opioid-sparing strategies, especially for high-volume interventions such as arthroscopic surgery, the most frequently performed orthopaedic procedure worldwide.^20,21^

Multimodal analgesia has gained attention as a strategy to reduce opioid reliance without compromising pain control. A recent randomized controlled trial (RCT) in the outpatient arthroscopy setting demonstrated significantly reduced opioid consumption with a multimodal regimen.^50^ Several systematic reviews have evaluated such strategies. A qualitative synthesis of adjunct analgesic techniques in hip arthroscopy showed reductions in early postoperative pain and opioid consumption, although it focused only on hip procedures and included invasive procedural analgesics.^34^ More recently, a systematic review found modest benefits with noninvasive adjuncts and cryopathy in hip, knee, and shoulder arthroscopy.^16^ However, neither review stratified outcomes by timing of administration, leaving the role of preoperative analgesia unclear. Supporting the relevance of this focus, another systematic review evaluated perioperative nonopioid analgesia in knee arthroscopy, finding that nonsteroidal anti-inflammatory drugs (NSAIDs), COX-2 inhibitors, and gabapentinoids reduced pain scores and opioid consumption within 24 hours postoperatively.^22^ In a subgroup of the 7 studies specifically assessing preoperative administration, opioid use was reduced by 11.8 mg in oral morphine equivalents (OMEs) and pain by 1.5 points on the visual analog scale (VAS).^18,22,29,38,44,47,57,58^

Despite these promising findings, the effectiveness of preoperative nonopioid analgesia remains inconsistently reported.^2,15,51^ Studies differ in analgesic class, timing, and control for confounders.^5,22,39,59^ Moreover, current evidence is predominantly knee focused, leaving knowledge gaps in other arthroscopic procedures.^2,43^ To address these limitations, a systematic review and meta-analysis of RCTs was conducted to evaluate the effect of preoperative nonopioid analgesia on postoperative opioid consumption and pain after arthroscopic surgery. Results were stratified by drug class and compared preoperative administration against peri- and postoperative approaches. Safety profiles and adverse effects were also evaluated. This synthesis of high-level evidence, across multiple surgery types, aims to inform clinical guidelines for optimizing analgesia while reducing postoperative opioid exposure.

## Methods

A systematic review and meta-analysis was conducted to evaluate the effectiveness of preoperative nonopioid analgesia in patients undergoing arthroscopic surgery. This review was performed following the PRISMA (Preferred Reporting Items for Systematic Reviews and Meta-analyses) guidelines and adhered to the methodological framework outlined by the Cochrane Collaboration for systematic reviews.^25,37^

### Eligibility Criteria

Inclusion and exclusion criteria were defined before the literature search to ensure the relevance and quality of included studies. Eligible studies were level 1 evidence RCTs that compared any method of preoperative nonopioid analgesia in adult patients undergoing primary arthroscopic surgery.^54^ Preoperative analgesia was defined as the administration of any nonopioid analgesic before surgery. The primary outcome of interest was postoperative opioid consumption, while secondary outcomes included postoperative pain scores and adverse events.

Studies conducted before 2005 were excluded to ensure the use of current medications and updated surgical protocols. Additional exclusion criteria included the use of nerve or spinal blocks and intra-articular injections. Revision arthroscopic procedures were excluded. Other excluded sources included non-English studies, textbook chapters, conference abstracts, and cadaveric or animal studies.

### Search Strategy

A comprehensive literature search on preoperative analgesia in arthroscopic surgery was performed on December 12, 2024, using PubMed, Embase, and MEDLINE. The search strategy included a combination of terms related to the surgical procedure, such as “arthroscopy,” “arthroscopic,” “meniscectomy,” “meniscal,” “meniscus,” and “anterior cruciate ligament,” as well as terms associated with analgesia and preoperative intervention, including “premedicate,” “preoperative,” “preventative,” “pre-emptive,” “analgesia,” and “pain.” No language restrictions were initially applied during the search. A detailed search strategy is available in Appendix Table A1 (available in the online version of this article).

### Study Selection

Two independent reviewers (J.D.-F. and R.F.) conducted a blinded duplicate review of all articles obtained through the systematic search. The title/abstract and full-text screening stages were performed in Covidence software.^69^ Conflicts at the title/abstract and full-text screening stages were resolved through consultation of senior authors (P.V. and D.C.).

Interreview reliability was assessed by the kappa statistic (κ), which quantifies the level of agreement between raters by comparing observed agreement while accounting for the agreement that would be expected by chance.^8^ The strength of agreement was classified as follows: κ = 0.81 to 1.0, almost perfect agreement; κ = 0.61 to 0.80, substantial agreement; κ = 0.41 to 0.60, moderate agreement; κ = 0.21 to 0.40, fair agreement; and κ ≤ 0.20, slight agreement.^36^

### Data Extraction

Data from the RCTs were extracted via Google Sheets (Google LLC). Extracted data included study characteristics (authors, year of publication, level of evidence), demographic data (sample size, age, body mass index), and surgical details (arthroscopic procedure performed and operative time). Details were extracted for the intervention (preoperative analgesic given, dose, route, and timing of medication) as well as postoperative outcomes (opioid consumption, pain scores, and adverse effects). The primary outcome of this study was the postoperative opioid consumption, converted to OME. Opioid consumption was recorded at 24 hours postoperatively, as well as at 48 hours and any additional time points reported in the studies. To ensure consistency, opioid consumption was converted to OME via a standardized conversion chart.^48^ Pain scores were recorded with the VAS as the standardized index instrument.^55^ Pain scores were recorded at 24 hours postoperatively. OME and VAS scores were reported as mean and standard deviation (SD) when available. Adverse events (eg, nausea, vomiting, headache, and dizziness) were reported as absolute case numbers and corresponding percentages.

### Missing Data

For studies that reported values in median and range, approximations were used to estimate the mean value and variance.^28^ If conversion was not feasible per the available data, SDs were calculated following guidelines from the *Cochrane Handbook for Systematic Reviews of Interventions*.^9,25^

### Statistical Analysis

Results were presented in a descriptive summary format. Means, ranges, percentages, and SDs were calculated via Google Sheets. If a study analyzed associated statistical parameters, *P* values were recorded. For postoperative opioid consumption and VAS scores, forest plots were generated by DataParty, with a DerSimonian and Laird random effects model. Heterogeneity was assessed with the *I*^2^ test. “Low,” “moderate,” and “high” values are identified by *I*^2^ values of 25% to 49%, 50% to 74%, and >75%, respectively.^26^ Forest plots were not generated for outcomes that did not have SDs widely reported across studies. Sensitivity analyses were conducted only if there were heavily influential studies, to minimize skewed outcomes. Statistical significance for differences was considered if *P* ≤ .05.

### Study Appraisal

Two independent reviewers (J.D.-F. and R.F.) assessed the overall quality of the RCTs using the revised Cochrane Risk of Bias 2 tool.^45,62^ Scores of high or low risk of bias or some concerns were assigned to each of the following domains: (1) bias arising from randomization, (2) bias attributed to deviations from intended interventions, (3) bias owing to missing outcome data, (4) bias in outcome measurement, and (5) bias in selection of reported results. Studies were categorized as follows: low risk of bias if all domains were rated low risk, high risk of bias if 1 or more domains were rated high risk, or some concerns otherwise. Any disagreements were resolved by a senior reviewer.

## Results

### Literature Search

**Figure 1.**
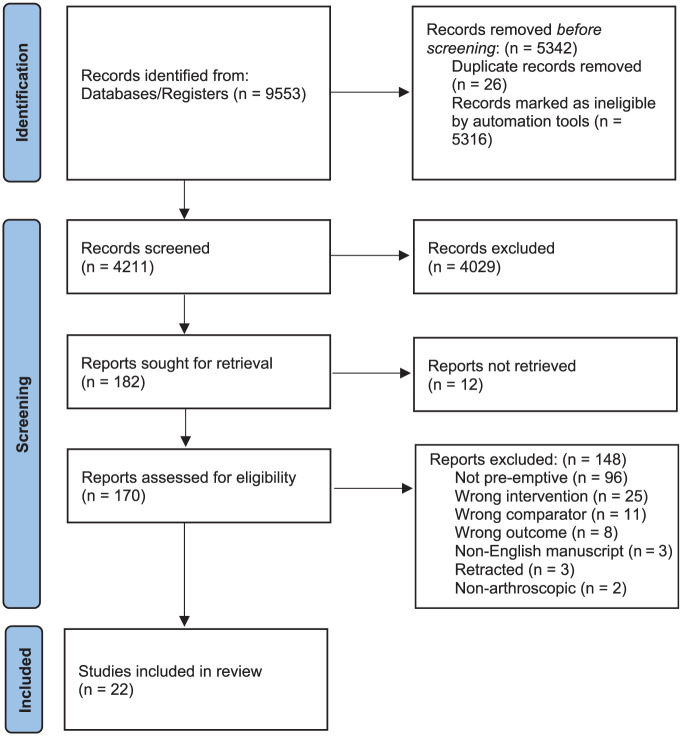
PRISMA (Preferred Reporting Items for Systematic Reviews and Meta-analyses) flow diagram representing a systematic review on preoperative analgesia for arthroscopic procedures.

The initial literature search yielded 9553 studies, of which 5342 duplicates were removed. Among the remaining 4211 unique articles, 4029 were removed after title and abstract screening. Systematic screening and assessment of eligibility yielded 22 full-text studies that satisfied inclusion criteria (Figure 1).^‖^

Interrater reliability analysis showed that substantial agreement was achieved at the title and abstract screening stage (κ = 0.71; 95% CI, 0.67-0.76) and full-text stage (κ = 0.77; 95% CI, 0.65-0.88). A risk of bias summary for the studies is provided in Appendix Figure A1 (available online).

### Study Characteristics

All the included studies were level 1 evidence. This review evaluated 22 RCTs that assessed the effectiveness of preoperative nonopioid pain medications for arthroscopic surgery. A total of 15, 4, and 3 studies assessed the effect of preoperative analgesia in arthroscopic knee,^¶^ hip,^13,30,74,76^ and shoulder^43,63,66^ surgery, respectively. Medications investigated included COX-2 inhibitors (14 studies),^#^ gabapentin (5 studies),^13,43,44,46,47^ pregabalin (2 studies),^32,49^ dextromethorphan (1 study),^18^ dexamethasone (1 study),^10^ and duloxetine (1 study).^63^ Of the 22 studies, 17 were blinded^**^ and 18 used a placebo control.^††^ A summary of study designs and protocols is provided in Appendix Table A2 (available online). Study characteristics and patient demographic information are found in Table 1.

**Table 1:** Study Characteristics*^a^*

First Author (Year)	Arthroscopic Procedure	Operative Time, min	Preoperative Intervention, Dose, Route	Timing/Nature of Preoperative Intervention	Sample Size	Female, %	Age, y	BMI, kg/m^2^
Boonriong (2010)^7^	ACL reconstruction	52.5 (10.8)	Etoricoxib (120 mg, PO)	1 dose 1 h PR	35	5.7	30 (NR)	NR
		60.7 (14.2)	Celecoxib (400 mg, PO)		35	11.4		
		60.5 (20.3)	Placebo		32	12.5		
Dahl (2012)^10^	ACL reconstruction	75 (17)	Parecoxib/valdecoxib (40 mg, IV) or etoricoxib (120 mg, PO)	1 dose 1 h PR	29	37.9	33 (NR)	NR
		75 (21)	Dexamethasone (8 mg, IV)		30	26.7	35 (NR)	
		71 (16)	Parecoxib/valdecoxib (40 mg, IV) or etoricoxib (120 mg, PO) + dexamethasone (8 mg, IV)		30	30	34 (NR)	
Degen (2023)^13^	Labral repair, osteochondroplasty, chondral debridement, labral debridement	NR	Control (oxycodone/acetaminophen, 5 mg/325 mg, PO)	1 to 2 doses every 6 h as needed	33	57.6	30.8 (9.1)	25.8 (4.6)
			Control + zopiclone (7.5 mg PO)	1 to 2 doses every 6 h as needed, zopiclone once nightly × 7 d	34	44.1	30 (9.9)	26.1 (4.7)
			Gabapentin (600 mg, PO)	1 dose 1 h PR	33	51.5	30.5 (10.3)	26.3 (4.7)
			Celecoxib (400 mg, PO)	1 dose 1 h PR	32	53.1	30.3 (8.9)	24.6 (3.7)
Ekman (2006)^17^	Meniscectomy/partial meniscectomy	NR	Celecoxib (400 mg, PO)	1 dose 1 h PR	80	46.5	45.5 (11.1)	NR
			Placebo		86	38.6	45 (10.9)	
Entezary (2013)^18^	Arthroscopic knee surgery	NR	Dextromethorphan (1 mg/kg PO)	1 dose 2-3 h PR	54	33.3	28.1 (8.6)	NR
			Placebo		58	36.2	30.2 (7.5)	
Hou (2019)^27^	Ligament reconstruction, meniscectomy, synovectomy, intra-articular fractures reduction	59 (9.1)	Meloxicam (7.5 mg PO)	2 doses 24 h and 1 dose 1 h PR	148	44.6	36.8 (7.6)	23.4 (3)
		58.5 (7.1)	Meloxicam (7.5 mg PO)	2 doses 4 h and 1 dose 24 h P/O	148	41.9	37.5 (7.5)	23.6 (3.2)
Kahlenberg (2017)^30^	Labral repair, labral debridement, labral reconstruction, acetabular osteoplasty, acetabular or femoral head chondroplasty	NR	Celecoxib (400 mg, PO)	1 dose 1 h PR	50	52	34.2 (NR)	NR
			Placebo		48	60.4	35.8 (NR)	
Kavak Akelma (2020)^32^	ACL reconstruction	80.3 (35.2)	Pregabalin (150 mg, PO)	1 dose 1 h PR	16	12.5	29.5 (9.5)	27.6 (3.9)
		75.5 (26.9)	Placebo		19	16.7	33.3 (14.1)	27.5 (5.7)
Lierz (2012)^38^	Arthroscopic knee surgery	38 (20)	Etoricoxib (120 mg, PO)	1 dose 1 h PR	33	60.6	54 (10)	28 (4)
		32 (14)	Placebo		33	60.6	56 (14)	27 (4)
Ma (2021)^41^	Knee ligament reconstruction, meniscectomy, synovectomy intra-articular fractures reduction	53.1 (8.6)	Celecoxib (200 mg, PO)	2 doses 2 h PR	100	53.9	40.4 (8.6)	23.2 (2.9)
			Meloxicam (7.5 mg, PO)	2 doses 2 h PR	71			
			Rofecoxib (25 mg, PO)	2 doses 2 h PR	61			
		52.4 (6.3)	Celecoxib (200 mg, PO)	2 doses 4 h; 1 dose at 12, 24, and 36 h P/O	108	56.5	41.5 (8.3)	23.3 (3.1)
			Meloxicam (7.5 mg, PO)	2 doses 4 h and 1 dose 24 P/O	65			
			Rofecoxib (25 mg, PO)	2 doses 4 h, 1 dose 24 h P/O	59			
Mardani-Kivi (2013)^42^	ACL reconstruction	40 (7)	Celecoxib (400 mg, PO)	1 dose 2 h PR	34	82.4	25.8 (7.7)	24 (2.6)
		36.7 (7)	Placebo		33	75.8	26.7 (4.9)	23.6 (3.5)
	Meniscectomy	30.3 (7)	Celecoxib (400 mg, PO)		31	71	32.7 (8)	24 (2.7)
		31.7 (4)	Placebo		32	62.5	32.2 (9.8)	23 (2.6)
Mardani-Kivi (2013)^44^	ACL reconstruction	40 (10)	Gabapentin (600 mg, PO)	1 dose 2 h PR	57	14	32.2 (9.3)	24 (2.2)
		36 (7)	Placebo		57	10.5	30.5 (10.2)	23.5 (2.8)
Mardani-Kivi (2016)^43^	Bankart repair	46.9 (10.7)	Gabapentin (600 mg, PO)	1 dose 2 h PR	38	2.8	30.2 (5)	23.3 (1.8)
		43.9 (9.5)	Placebo		38	2.1	28.3 (4.4)	24.1 (3.4)
Ménigaux (2005)^46^	ACL reconstruction	81 (34)	Gabapentin (1200 mg, PO)	1 dose 1-2 h PR	20	30	31 (8)	NR
		79 (23)	Placebo		20	35	31 (8)	
Montazeri (2007)^47^	Arthroscopic knee surgery	105.1 (7.2)	Gabapentin (300 mg, PO)	1 dose 2 h PR	35	25.7	34.7 (18.1)	NR
		103.3 (6.4)	Placebo		35	20	34.6 (17.8)	
Nimmaanrat (2012)^49^	ACL reconstruction	100.7 (NR)	Pregabalin (75 mg, PO)	1 dose 1 h PR	27	21.1	29.3 (NR)	23.4 (NR)
		92.6 (NR)	Placebo		29	20.3	33.8 (NR)	23.7 (NR)
Su (2022)^63^	Rotator cuff repair capsular release, bicep tenotomy/tenodesis, distal clavicle resection	NR	Duloxetine (60 mg, PO)	1 dose at 48 h and 24 h PR	60	50	55.8 (7.6)	25.2 (4.6)
			Placebo		60	53.3	56.5 (8)	26 (4.2)
Toivonen (2007)^66^	Acromioplasty	45 (15)	Etoricoxib (120 mg, PO)	1 dose 1 h PR	15	26.7	52 (10)	27 (4)
		49 (17)	Placebo		15	20	52 (7)	27 (3)
Uribe (2018)^67^	Arthroscopic knee surgery	27.3 (14.5)	Ibuprofen (800 mg, IV)	1 dose 2 h PR	20	30	42.3 (12.4)	NR
		25.5 (25.6)	Ketorolac (30 mg, PO)	1 dose immediately P/O	31	32.3	44.6 (13)	
Zhang (2014)^74^	Arthroscopic hip surgery	67 (7)	Celecoxib (200 mg, PO)	1 dose 1 h PR	27	48.1	41 (4.9)	33 (5.1)
		90 (3)	Placebo		26	57.7	43.5 (5.1)	35 (4.9)
Zhou (2017)^75^	Meniscectomy/partial meniscectomy	60.4 (8.8)	Celecoxib (400 mg, PO)	1 dose 24 h and 12 h PR	68	35.3	34.7 (7.1)	23.9 (2.8)
		59.9 (7.8)		1 dose 1 h PR	69	50.7	36 (6.1)	23.4 (3.2)
		56.7 (7.1)	Celecoxib (200 mg, PO)	2 doses 4 h and 1 dose 16 h postoperatively P/O	69	40.6	35.9 (6.6)	24.2 (3.3)
Zhu (2020)^76^	Arthroscopic hip surgery	NR	Celecoxib (200 mg PO)	2 doses 24 h, 1 dose 12 h and 2 h PR	50	46	34.3 (6.2)	NR
				2 doses at 12 h P/O, then 1 dose twice daily until POD 7	50	52	35.5 (7.1)	

a Data are presented as mean (SD) unless noted otherwise. ACL, anterior cruciate ligament; BMI, body mass index; IV, intravenous; NR, no result; P/O, postoperative; PO, per os (oral); PR, preoperative.

### Postoperative Opioid Consumption and Pain Scores

**Figure 2.**
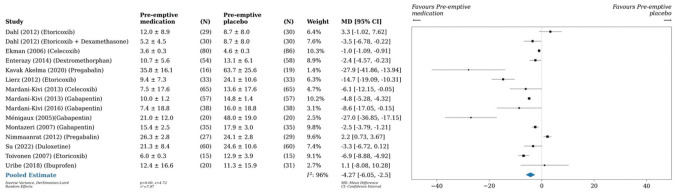
Forest plot (random effects) shows a reduction in cumulative opioid consumption by 4.3 mg (95% CI, –6.1 to –2.5; *P* < .001; *I*^2^ = 96%) 24 hours postoperatively in patients taking any preoperative medication as compared with placebo. MD, mean difference.

**Figure 3.**
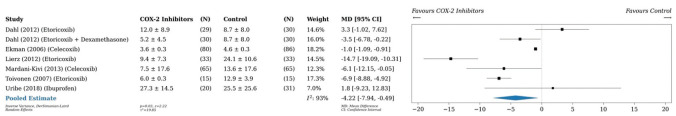
Forest plot (random effects) shows a reduction in cumulative opioid consumption by 4.2 mg (95% CI, –7.9 to –0.5; *P* = .03; *I*^2^ = 93%) 24 hours postoperatively in patients taking COX-2 inhibitors as compared with placebo. MD, mean difference.

**Figure 4.**
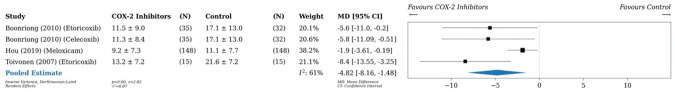
Forest plot (random effects) shows a reduction in cumulative opioid consumption by 4.8 mg (95% CI, –8.2 to –1.5; *P* < .001; *I*^2^ = 61%) 48 hours postoperatively in patients taking COX-2 inhibitors as compared with placebo. MD, mean difference.

**Figure 5.**
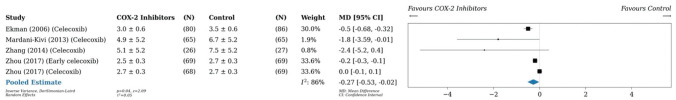
Forest plot (random effects) shows a reduction in postoperative visual analog scale scores for pain by 0.3 (95% CI, –0.5 to –0.02; *P* = .04; *I*^2^ = 86%) 24 hours postoperatively in patients taking COX-2 inhibitors as compared with placebo. MD, mean difference.

**Figure 6.**
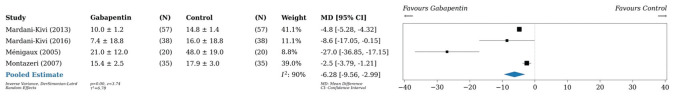
Forest plot (random effects) shows a reduction in cumulative opioid consumption by 6.3 mg (95% CI, –9.6 to –3.0; *P* < .001; *I*^2^ = 90%) 24 hours postoperatively in patients taking gabapentin as compared with placebo. MD, mean difference.

A pairwise meta-analysis was conducted on 14 studies (n = 1185),^‡‡^ which found a reduction in postoperative opioid consumption by 4.3 mg (95% CI, –6.1 to –2.5; *P* < .001; *I*^2^ = 96%) 24 hours postoperatively in patients taking any preemptive medication as compared with placebo (Figure 2). A pairwise meta-analysis conducted on 6 studies (n = 562)^10,17,38,42,66,67^ and another on 3 studies (n = 610)^7,27,66^ demonstrated that preoperative analgesia with COX-2 inhibitors reduced cumulative opioid consumption versus placebo by 4.2 mg (95% CI, –7.9 to –0.5; *P* = .03; *I*^2^ = 93%) 24 hours postoperatively and by 4.8 mg (95% CI, –8.2 to –1.5; *P* < .001; *I*^2^ = 61%) 48 hours postoperatively, respectively (Figures 3 and 4). Similarly, preoperative administration of COX-2 inhibitors reduced VAS pain scores by 0.3 (95% CI, –0.5 to –0.02; *P* = .04; *I*^2^ = 86%) 24 hours postoperatively as compared with placebo (Figure 5).^17,42,74,75^ A pairwise meta-analysis conducted on 4 studies (n = 300) found a reduction in OME by 6.3 mg (95% CI, –9.6 to –3.0; *P* < .001; *I*^2^ = 90%) 24 hours postoperatively in patients taking gabapentin as compared with placebo (Figure 6).^43,44,46,47^

**Figure 7.**
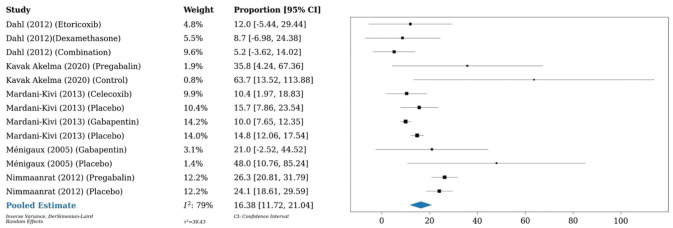
Forest plot (random effects) shows an oral morphine equivalent of 16.4 mg (95% CI, 11.7-21; *I*^2^ = 79%) 24 hours postoperatively in patients who underwent anterior cruciate ligament reconstruction.

**Figure 8.**
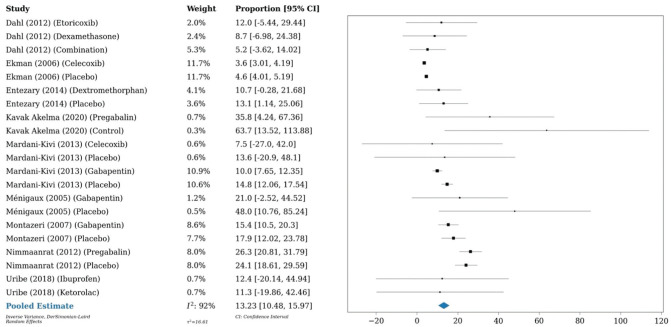
Forest plot (random effects) shows an oral morphine equivalent of 13.2 mg (95% CI, 10.5-16; *I*^2^ = 92%) 24 hours postoperatively in patients who underwent any knee arthroscopy.

**Figure 9.**
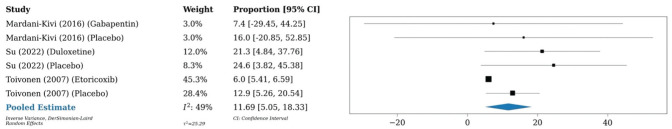
Forest plot (random effects) shows an oral morphine equivalent of 11.7 mg (95% CI, 5.1-18.3; *I*^2^ = 49%) 24 hours postoperatively in patients who underwent shoulder arthroscopy.

A 1-arm meta-analysis conducted on 6 studies (n = 401) demonstrated an OME of 16.4 mg at 24 hours postoperatively in patients who underwent anterior cruciate ligament reconstruction, as compared with a meta-analysis of 10 studies (n = 863) that showed an OME of 13.2 mg in those who underwent any type of knee arthroscopy. A meta-analysis of 3 studies (n = 226) investigating any type of shoulder arthroscopy showed an OME of 11.7 mg at 24 hours postoperatively (Figures 7-9).

Four studies^27,30,75,76^ compared the effects of administering the same nonopioid analgesic medication pre- or postoperatively, all of which investigated COX-2 inhibitors. A quantitative statistical analysis was not performed because of variability in timing and reporting. However, all these studies found a greater reduction in postoperative opioid consumption in patients treated with preoperative administration of COX-2 inhibitors as compared with postoperative administration; these relationships were statistically significant in 3 (75%) studies.^27,41,76^

Two studies investigated the effect of administering different nonopioid medications pre- and postoperatively.^13,67^ One study compared preoperative gabapentin or celecoxib against control (oxycodone-acetaminophen + naproxen [Naprosyn]) or postoperative zopiclone,^13^ and the other compared preoperative ibuprofen administration against postoperative ketorolac^67^; both studies found no statistically significant differences in postoperative opioid consumption or pain scores between groups. An overview of outcomes is provided in Table 2.

**Table 2:** Postoperative Outcomes and Adverse Events*^a^*

		OME Consumption			VAS			
First Author (Year)	Intervention	Postoperative Time Point	Total Rescue, mg	Time to First Dose of First Analgesia Requirement, min	Postoperative Time Point	Score	Time to Discharge, min	Adverse Events, No. (%)
Boonriong (2010)^7^	PR etoricoxib	48 h	11.5 (9)	353.4 (842.4)	—	NR	2880 (NR)	Dyspepsia: 1 (2.9), nausea: 1 (2.9), dizziness: 3 (8.6), tachycardia: 2 (5.7), hypertension: 3 (8.6), fever: 2 (5.7)
	PR celecoxib		11.3 (8.4)	349.8 (613.2)				Dizziness: 2 (5.7), headache: 1 (2.9), fever: 12 (34.3)
	PR placebo		17.1 (13)	300 (351)				Dyspepsia: 2 (6.3), flatulence: 1 (3.1), vomiting: 2 (6.3), constipation: 3 (9.4), dizziness: 4 (12.5), headache: 2 (6.3), tachycardia: 3 (9.4), hypertension: 5 (15.6), oliguria: 1 (3.1), fever: 11 (34.4)
Dahl (2012)^10^	PR parecoxib- valdecoxib- etoricoxib	24 h	12 (8.9)	NR	—	NR	161 (50)	PONV (24 h): 6 (20.7)
	PR dexamethasone		8.7 (8)				190 (60)	PONV (24 h): 7 (23.3)
	PR combination		5.2 (4.5)				166 (67)	PONV (24 h): 5 (16.7)
Degen (2023)^13^	P/O control	—	NR	NR	—	NR	155.7	Acute pain exacerbation: 1 (3)
	P/O control + zopiclone		NR				165.8	Pulmonary embolism: 1 (3), metallic taste: 1 (3), superficial infection: 1 (3)
	PR gabapentin		NR				149.0	Acute pain exacerbation: 5 (15.2), PONV: 1 (3), deep vein thrombosis: 1 (3)
	PR celecoxib		NR				156.3	Acute pain exacerbation: 2 (6.3), PONV: 1 (3.1)
Ekman (2006)^17^	PR celecoxib	24 h	3.6 (0.3)	NR	24 h	3 (0.6)	NR	Nausea: 1 (1.3)
	PR placebo		4.6 (0.3)			3.5 (0.6)		Headache: 2 (2.3), nausea: 1 (1.1), constipation: 1 (1.1), pulmonary embolism: 1 (1.1), myalgia: 1 (1.1), pruritus: 1 (1.1)
Entezary (2013)^18^	PR dextromethorphan	24 h	10.7 (5.6)	222 (78)	PACU	4.1 (1.8)	NR	PONV: 13 (24.1), itching: 10 (18.5)
	PR placebo		13.1 (6.1)	186 (72)	PACU	4.7 (1.2)		PONV: 23 (39.7), itching: 12 (20.7)
Hou (2019)^27^	PR meloxicam	48 h	9.2 (7.3)	NR	—	NR	NR	Nausea 32 (21.6), constipation: 28 (18.9), vomiting: 14 (9.5), dizziness: 8 (5.4), drowsiness: 5 (3.4)
	P/O meloxicam		11.1 (7.7)					Nausea 38 (25.7), constipation: 27 (18.2), vomiting: 13 (8.8), dizziness: 9 (6.1), drowsiness: 5 (3.4)
Kahlenberg (2017)^30^	PR celecoxib	PACU	15.3 (0.2)	NR	PACU	4.4 (1.5)	153.0	None
	PR placebo		15.4 (0.2)			5.0 (1.5)	173.0	
Kavak Akelma (2020)^32^	PR pregabalin	24 h	35.8 (16.1)	386.3 (47.6)	—	NR	NR	PONV: 2 (12.5), drowsiness: 1 (6.3), urinary retention: 1 (6.3)
	PR control		63.7 (25.6)	272.8 (45.2)				PONV: 2 (10.5), urinary retention: 1 (5.3)
Lierz (2012)^38^	PR etoricoxib	24 h	9.4 (7.3)	120 (NR)	—	NR	2820 (180)	Nausea (1), shivering (1)
	PR placebo		24.1 (10.6)	0 (NR)				Nausea (5), dizziness (3), vomiting (1), hypotension (1)
Ma (2021)^41^	PR celecoxib	Until discharge	10.8 (5.2)	NR	—	NR	NR	Nausea: 16 (16), vomiting: 4 (4), constipation: 12 (12), drowsiness: 2 (2), dizziness: 1 (1)
	PR meloxicam		11.8 (5.9)					Nausea: 12 (16.9), vomiting: 4 (5.6), constipation: 9 (12.7), drowsiness: 1 (1.4), dizziness: 1 (1.4)
	PR rofecoxib		11.0 (6.0)					Nausea: 5 (8.2), vomiting: 2 (3.3), constipation: 7 (11.5), drowsiness: 1 (1.6), dizziness: 0 (0)
	P/O celecoxib		12.0 (7.3)					Nausea: 15 (13.9), vomiting: 5 (4.6), constipation: 9 (8.3), drowsiness: 2 (1.9), dizziness: 3 (2.8)
	P/O meloxicam		13.8 (6.6)					Nausea: 20 (30.8), vomiting: 5 (7.7), constipation: 9 (13.8), drowsiness: 2 (3.1), dizziness: 2 (3.1)
	P/O rofecoxib		13.2 (12.6)					Nausea: 12 (20.3), vomiting: 3 (5.1), constipation: 14 (23.7), drowsiness: 2 (3.4), dizziness: 1 (1.7)
Mardani-Kivi (2013)^42^	PR celecoxib	24 h	7.5 (17.6)	NR	24 h	4.9 (5.2)	NR	Nausea: 12 (21.1), vomiting: 2 (3.5), dizziness: 2 (3.5), drowsiness: 2 (3.5)
	PR placebo		13.6 (17.6)			6.7 (5.2)		Nausea: 21 (35), dizziness: 4 (6.7), drowsiness: 2 (3.3), vomiting: 2 (3.3)
Mardani-Kivi (2013)^44^	PR gabapentin	24 h	10 (1.2)	NR	24 h	4.4 (NR)	NR	Nausea: 3 (5.4), dizziness: 3 (5.4)
	PR placebo		14.8 (1.4)			NR		Dizziness: 6 (11), nausea: 4 (7.5)
Mardani-Kivi (2016)^43^	PR gabapentin	24 h	7.4 (18.8)	NR	24 h	4.7 (1.3)	NR	Dizziness: 5 (14), nausea: 1 (3)
	PR placebo		16 (18.8)			5.3 (1.3)		Dizziness: 3 (9), nausea: 2 (6)
Ménigaux (2005)^46^	PR gabapentin	24 h	21 (12)	16 (28)	—	NR	NR	Nausea: 3 (15)
		48 h	29 (22)					
	PR placebo	24 h	48 (19)	1 (2)				Nausea: 3 (15)
		48 h	69 (40)					
Montazeri (2007)^47^	PR gabapentin	24 h	15.4 (2.5)	31.6 (15.9)	24 h	4.5 (1.8)	NR	Nausea: 6 (17.1), vomiting: 4 (11.4), fatigue 1 (2.9), light-headedness: 1 (2.9), dizziness: 1 (2.9)
	PR placebo		17.9 (3)	26.7 (7.1)		6.7 (2.6)		Nausea: 5 (14.3), vomiting: 3 (8.6)
Nimmaanrat (2012)^49^	PR pregabalin	24 h	26.3 (2.8)	NR	—	NR	NR	PONV: 10 (37), dizziness: 7 (25.9), headache: 6 (22.4), blurred vision: 2 (7.4)
	PR placebo		24.1 (2.8)					PONV: 14 (48.2), dizziness: 14 (48.2), headache: 7 (24.1), blurred vision: 3 (10.3)
Su (2022)^63^	PR duloxetine	24 h	21.3 (8.4)	NR	—	NR	NR	PONV: 16 (26.7), drowsiness: 7 (11.7), insomnia: 5 (8.3), dry mouth: 4 (6.7), dizziness: 3 (5), constipation: 3 (5), fatigue: 2 (3.3)
		48 h	3.4 (7.7)					
	PR placebo	24 h	24.6 (10.6)					PONV: 6 (10), insomnia: 6 (10), dizziness: 5 (8.3), drowsiness: 3 (5), dry mouth: 2 (3.3), constipation: 2 (3.3), fatigue: 2 (3.3)
		48 h	4.2 (7.3)					
Toivonen (2007)^66^	PR etoricoxib	24 h	6 (0.3)	NR	—	NR	91 (23)	Before discharge—drowsiness: 2 (13.3), nausea: 2 (13.3). After discharge— nausea: 1 (6.7), headache: 1 (6.7)
		48 h	13.2 (7.2)					
	PR placebo	24 h	12.9 (3.9)				128 (50)	Before discharge—drowsiness: 6 (40), nausea: 3 (20). After discharge— drowsiness 4 (26.7), nausea: 1 (6.7), headache: 2 (13.3), difficulty to void: 1 (6.7), constipation: 1 (6.7)
		48 h	21.6 (7.2)					
Uribe (2018)^67^	PR ibuprofen	24 h	12.4 (16.6)	77.6 (33.0)	—	NR	NR	NR
	PR ketorolac		11.3 (15.9)	55.8 (35.4)				
Zhang (2014)^74^	PR celecoxib	—	NR	NR	PACU	7.2 (0.7)	NR	NR
					24 h	5.1 (5.2)		
	PR placebo	—			PACU	7.5 (0.7)		
					24 h	7.5 (5.2)		
Zhou (2017)^75^	Early PR celecoxib	—	NR	NR	24 h	2.5 (0.3)	NR	NR
	PR celecoxib					2.7 (0.3)		
	P/O celecoxib					2.7 (0.3)		

a Data are presented as mean (SD) unless noted otherwise. NR, no result; OME, oral morphine equivalent; PACU, postanesthesia care unit; P/O, postoperative; PONV, postoperative nausea and vomiting PR, preoperative; VAS, visual analog scale.

### Adverse Events

Eighteen studies reported postoperative complications. Pairwise meta-analyses of 8 studies (n = 734) and 6 studies (n = 460) showed no significant differences in postoperative rates of nausea or nausea and vomiting, respectively (Appendix Figures A2 and A3, available online). A pairwise meta-analysis conducted on 4 studies (n = 254) showed that preoperative administration of nonopioid analgesics was associated with a significantly higher risk of postoperative drowsiness as compared with placebo (Appendix Figure A4, available online). Further quantitative analysis was limited owing to variability in data reporting. There were 3 major adverse events: 2 pulmonary embolisms^13,17^ and 1 deep vein thrombosis.^13^ These major adverse events were not directly correlated to nonopioid medication use. The most common adverse events in the studies were nausea and vomiting. Statistically significant differences in complications between groups were reported in only 4 studies.^7,41,43,63^ Long-term complications were not available given the short follow-up period of studies. Full details are found in Table 2.

## Discussion

The primary finding of this study is that preoperative analgesia with nonopioid medications for patients undergoing arthroscopic procedures results in a statistically significant reduction in opioid consumption at 24 hours postoperatively as compared with placebo. To enhance clinical interpretability, reductions in opioid consumption from OMEs were converted to oral oxycodone equivalents according to a standard conversion ratio of 1.5:1.^1^ In the pooled analysis, preoperative administration of any nonopioid analgesic resulted in a mean reduction of 4.3 mg OME at 24 hours, corresponding to approximately 2.9 mg of oral oxycodone. Subgroup analysis of COX-2 inhibitors showed comparable opioid-sparing effects, with oral oxycodone reductions of 2.8 mg at 24 hours and 3.2 mg at 48 hours postoperatively as compared with placebo. Gabapentin demonstrated the largest reduction among the evaluated interventions, with a pooled decrease of 4.2 mg of oral oxycodone. Despite the statistical significance of these results, these reductions may not be clinically impactful. Preoperative administration of COX-2 inhibitors also led to statistically significant reductions in postoperative pain scores as compared with placebo. Similarly, while this reduction reached statistical significance, it may not be clinically meaningful. When converted to a 100-mm scale, the difference in VAS scores corresponds to 3 mm. Previous studies across arthroscopic populations and acute pain settings report minimum clinically important difference values for VAS scores, ranging from 8 to 40 mm, depending on the context and methodology. As such, the observed effect size likely falls below the threshold for clinical significance, and this finding should be interpreted with caution.^53^ Furthermore, greater reductions in postoperative opioid consumption were achieved when the same COX-2 inhibitor was administered preoperatively as compared with postoperatively. Given these promising findings, it is crucial to investigate the utility of different preoperative medications to help guide perioperative protocols with the goal of minimizing the use of opioids after arthroscopy.

The most investigated preoperative medication types were selective COX-2 inhibitors and nonselective NSAIDs across the studies in this review, followed by gabapentin. The preoperative administration of gabapentin led to a greater treatment effect on postoperative opioid consumption as compared with COX-2 inhibitors. In addition, preoperative administration of COX-2 inhibitors was associated with a greater reduction in opioid consumption as compared with postoperative intervention. Across all studies, only 1 major adverse event was reported from patients taking gabapentin, whereas none were reported with COX-2 inhibitors. Importantly, there was considerable variability across the studies in terms of type, dosage, and timing of these medications. Additionally, the type of surgery varied widely across studies, which likely contributed to the heterogeneity in postoperative opioid requirements. To explore this, subgroup analyses were conducted by surgical procedure. Patients undergoing anterior cruciate ligament reconstruction exhibited the highest mean opioid consumption at 24 hours postoperatively (16.4-mg OME), as compared with those undergoing any type of knee arthroscopy (13.2-mg OME) or shoulder arthroscopy (11.7-mg OME). This gradient likely reflects the greater tissue trauma and postoperative pain associated with ligament reconstruction relative to simpler diagnostic or debridement procedures. While these findings suggest that procedural complexity may influence the efficacy or opioid-sparing effect of preoperative analgesia, the limited number of studies and inconsistent reporting at later postoperative time points precluded additional subgroup analyses.

Variability in medication type, dosage and timing, and surgical procedure likely contributed to the high heterogeneity observed across studies, reflected by an *I*^2^ > 75% for outcomes measured at 24 hours postoperatively. While these findings are consistent with those of a systematic review conducted in 2020, which demonstrated similarly high heterogeneity values, the treatment effects observed in the present study were smaller by comparison.^22^ Improved perioperative pain control as a result of recent advancements in modern multimodal pain management and surgical techniques likely contributes to this discrepancy.^68,73^ Unlike the previous review, which examined perioperative analgesia broadly—including pre-, intra-, and postoperative interventions—the current study focuses on the role of preoperative nonopioid analgesia.^18^ By isolating the effect of preoperative analgesia, this review addresses a distinct and clinically relevant gap in the literature, offering targeted guidance for perioperative pain protocols aimed at minimizing opioid use after arthroscopy.

To critically evaluate the clinical utility of preoperative analgesia, the benefits of reducing opioid consumption and improving pain scores must be weighed against the possible side effects and risks associated with different nonopioid medications. Although NSAIDs are given as only a one-time dose in the setting of preoperative analgesia, it is important to acknowledge that long-term use of nonselective NSAIDs is associated with a range of upper and lower gastrointestinal (GI) adverse effects, including but not limited to dyspepsia, gastric erosion and ulcers, and GI bleeding. Furthermore, NSAIDs are associated with an increased risk for hypertension, acute renal failure, and heart failure exacerbation.^35,61,70,72^ Selective COX-2 inhibitors were developed with the goal of minimizing GI symptoms; however, they have been shown to increase the risk of cardiovascular events.^12,33,40,72^ Gabapentin has been associated with a range of adverse effects, including teratogenicity and neurological, behavioral, and respiratory-related complications.^52,56^ Yet, these complications were not observed in this study, indicating that these adjunct medications seem to be well tolerated with strong safety profiles in clinical practice. Moreover, the quantitative analyses indicate that patients treated with nonopioid medication preoperatively did not experience significant differences in nausea or vomiting as compared with placebo. Regardless, these medications should be prescribed cautiously after an in-depth conversation between the patient and physician regarding potential side effects, and their use should be avoided in certain at-risk populations.

Opioids are routinely overprescribed after arthroscopy, and patients frequently have a surplus of pills once recovered from surgery.^60,65,71^ A cohort study reported that 80% of opioid-naive patients filled a prescription for opioids after knee arthroscopy. Furthermore, 2 studies found that >75% of patients stopped taking opioids by the fifth postoperative day after knee arthroscopy.^65,71^ Various studies have shown that satisfactory pain control after arthroscopic procedures can be achieved with minimal or no opioid consumption.^11,24,31^ Given the common practice of overprescription, particularly after arthroscopic surgery, it is evident that orthopaedic surgeons have a considerable role to play in curbing the opioid epidemic.

The isolated effects of preoperative analgesia described in this review are promising. However, it is important to recognize that optimal perioperative pain management will be achieved only with a holistic approach tailored to each patient’s unique needs. For example, a previous systematic review reports the benefits of pre- and/or postoperative intervention with nonopioid medications, as well as single-injection nerve blocks and cryotherapy for postoperative pain relief. These authors also discuss the reductions in postoperative opioid consumption and pain associated with preoperative patient education, which is a well-established finding in arthroscopic studies.^3,22,64^ Further research is needed to clarify how to achieve optimal perioperative pain management for different patient populations.

The primary limitation of this review is due to the considerable variability in medication type, dosage, and timing, leading to high statistical heterogeneity. The high heterogeneity associated with each meta-analysis introduces a significant risk of bias, which hinders the precision of this article’s findings. Furthermore, differences in outcomes and postoperative time points at which these outcomes were measured limited the ability to conduct additional statistical analyses. Given the heterogeneity in interventions and outcomes, a network meta-analysis could not be conducted to compare multiple preoperative medications. In addition, several of the studies did not report on specific statistics that would increase the significance of the findings, such as time to first dose of analgesia or number of opioid refill requests. Finally, the majority of studies did not measure postoperative outcomes after 48 hours postoperatively; thus, the long-term use of opioids after arthroscopy was not evaluated in this review. This comprehensive systematic review evaluating the effect of preoperative analgesia for patients undergoing arthroscopic procedures is the most recent and updated assessment of the literature. The major strength of this review is due to the high quality of evidence of studies, all of which were RCTs. It is difficult to directly compare different types and dosages of medications owing to potential confounding effects, emphasizing the importance of conducting high-quality comparative studies. This review recommends conducting a series of multicenter prospective studies with standardized protocols for dosage and timing of intervention, as well as standardized follow-up protocols to more accurately evaluate key outcomes.

## Conclusion

This systematic review demonstrated that preoperative treatment with nonopioid medications, particularly COX-2 inhibitors and gabapentin, is associated with statistically significant reductions in postoperative opioid consumption after arthroscopic surgery. Despite significant statistical findings, observed reductions in postoperative opioid consumption and VAS pain scores may not represent clinically meaningful improvements. The current available literature is highly heterogeneous, indicating the need for high-quality prospective studies to more accurately assess optimal approaches to pain management.

## Supplemental Material

sj-pdf-1-ajs-10.1177_03635465251396164 – Supplemental material for Preoperative Nonopioid Analgesia Reduces Postoperative Opioid Consumption After Arthroscopic Surgery: A Systematic Review and Meta-analysis of Randomized Controlled Trials Supplemental material, sj-pdf-1-ajs-10.1177_03635465251396164 for Preoperative Nonopioid Analgesia Reduces Postoperative Opioid Consumption After Arthroscopic Surgery: A Systematic Review and Meta-analysis of Randomized Controlled Trials by Joshua Dworsky-Fried, Ryley Fowler, Prushoth Vivekanantha, Dan Cohen, Nicole Simunovic, Darren de SA and Olufemi R. Ayeni in The American Journal of Sports Medicine

## Footnotes

‖ References 7, 10, 13, 17, 18, 27, 30, 32, 38, 41-44, 46, 47, 49, 63, 66, 67, 74-76

¶ References 7, 10, 17, 18, 27, 32, 38, 41, 42, 44, 46, 47, 49, 67, 75

# References 7, 10, 13, 17, 27, 30, 38, 41, 42, 66, 67, 74-76

** References 7, 10, 13, 17, 18, 30, 32, 38, 42-44, 46, 47, 63, 66, 67, 74

†† References 7, 10, 13, 17, 18, 30, 32, 38, 42-44, 46, 47, 49, 63, 66, 67, 74

‡‡ References 10, 17, 18, 32, 38, 42-44, 46, 47, 49, 63, 66, 67
