# Supplementary material for: Preoperative Nonopioid Analgesia Reduces Postoperative Opioid Consumption After Arthroscopic Surgery: A Systematic Review and Meta-analysis of Randomized Controlled Trials
Source: Am J Sports Med. 2026 Jan 13;54(6):1509–24. doi: 10.1177/03635465251396164 (PMC13133421; doi:10.1177/03635465251396164)
Supplement: sj-pdf-1-ajs-10.1177_03635465251396164 – Supplemental material for Preoperative Nonopioid Analgesia Reduces Postoperative Opioid Consumption After Arthroscopic Surgery: A Systematic Review and Meta-analysis of Randomized Controlled Trials [file sj-pdf-1-ajs-10.1177_03635465251396164.pdf]

Preoperative non-opioid analgesia reduces postoperative opioid consumption following arthroscopic surgery: a systematic review and meta-analysis of randomized controlled trials

APPENDIX

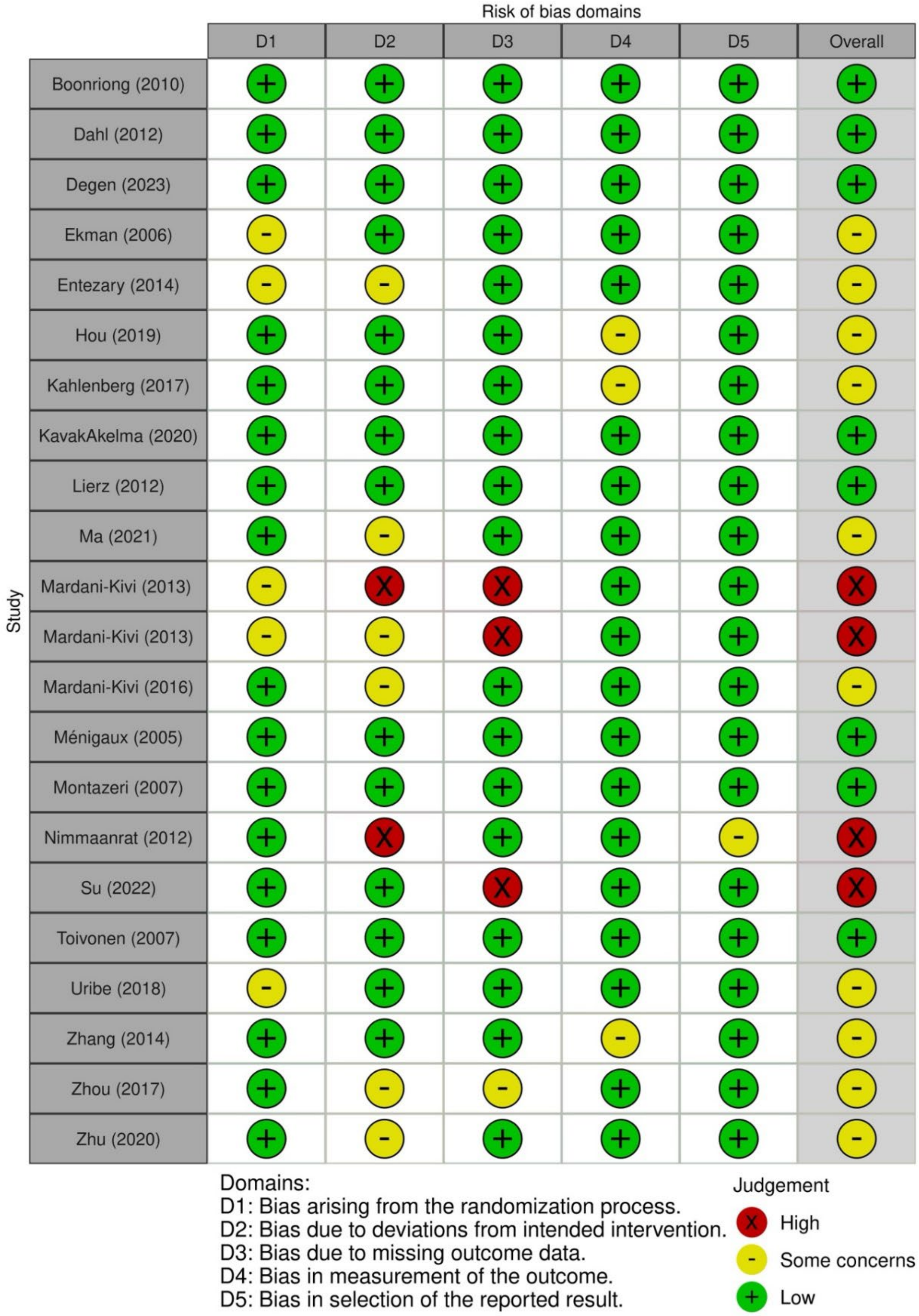

Figure A1. Risk of bias summary

Study	Comparator	(%)	Control	(%)	Weight	RR [95% CI]
Boonriong (2010) (Etoricoxib)	1/35	(2.9%)	0/32	(0.0%)	2.1%	2.75 [0.12, 65.18]
Degen (2023) (Gabapentin)	1/33	(3.0%)	0/33	(0.0%)	2.1%	3.0 [0.13, 71.08]
Degen (2023) (Celecoxib)	1/32	(3.1%)	0/33	(0.0%)	2.1%	3.09 [0.13, 73.19]
Ekman (2006) (Celecoxib)	1/99	(1.0%)	1/101	(1.0%)	2.8%	1.02 [0.06, 16.09]
Lierz (2012) (Etoricoxib)	1/33	(3.0%)	5/33	(15.2%)	4.9%	0.2 [0.02, 1.62]
Mardani-Kivi (2013) (Celecoxib)	12/65	(18.5%)	21/65	(32.3%)	55.3%	0.57 [0.31, 1.06]
Ménigaux (2005) (Gabapentin)	3/20	(15.0%)	3/20	(15.0%)	9.8%	1.0 [0.23, 4.37]
Montazeri (2007) (Gabapentin)	6/35	(17.1%)	5/35	(14.3%)	17.9%	1.2 [0.4, 3.57]
Toivonen (2007)(Etoricoxib)	1/15	(6.7%)	1/15	(6.7%)	3.0%	1.0 [0.07, 14.55]
**Pooled Estimate**					I^2: 0%	0.75 [0.47, 1.19]

Mantel-Haenszel, DerSimonian-Laird Random Effects — *p=0.22, z=1.21, τ^2=0.00* — *RR: Risk Ratio, CI: Confidence Interval*

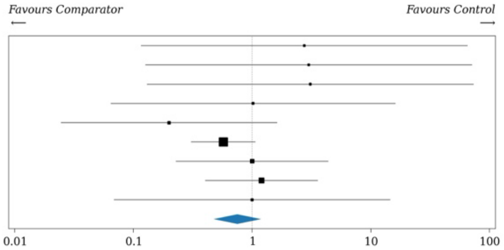

Figure A2. Forest plot (random effects) showing comparing rates of postoperative nausea between pre-emptive medications and placebo

Study	Comparator	(%)	Control	(%)	Weight	RR [95% CI]
Dahl (2012) (Parecoxib)	6/29	(20.7%)	7/30	(23.3%)	19.9%	0.89 [0.34, 2.32]
Kavak Akelma (2020) (Pregabalin)	2/16	(12.5%)	2/19	(10.5%)	6.6%	1.19 [0.19, 7.51]
Mardani-Kivi (2013) (Gabapentin)	3/57	(5.3%)	4/57	(7.0%)	10.2%	0.75 [0.18, 3.2]
Mardani-Kivi (2016) (Gabapentin)	1/38	(2.6%)	2/38	(5.3%)	4.2%	0.5 [0.05, 5.28]
Nimmaanrat (2012) (Pregabalin)	10/27	(37.0%)	14/29	(48.3%)	35.8%	0.77 [0.41, 1.43]
Su (2022) (Duloxetine)	16/60	(26.7%)	6/60	(10.0%)	23.3%	2.67 [1.12, 6.35]
**Pooled Estimate**					I^2: 20%	1.06 [0.65, 1.75]

Mantel-Haenszel, DerSimonian-Laird Random Effects — *p=0.80, z=0.25, τ^2=0.08* — *RR: Risk Ratio, CI: Confidence Interval*

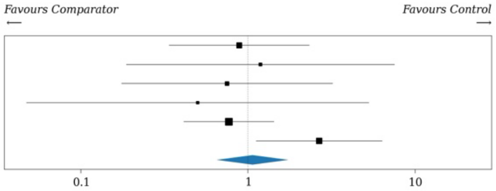

Figure A3. Forest plot (random effects) showing comparing rates of postoperative nausea and vomiting between pre-emptive medications and placebo

Study	Comparator	(%)	Control	(%)	Weight	RR [95% CI]
Kavak Akelma (2020) (Pregabalin)	1/16	(6.2%)	0/18	(0.0%)	11.2%	3.35 [0.15, 76.93]
Montazeri (2007) (Gabapentin)	1/35	(2.9%)	0/35	(0.0%)	10.9%	3.0 [0.13, 71.22]
Su (2022) (Duloxetine)	7/60	(11.7%)	3/60	(5.0%)	64.3%	2.33 [0.63, 8.6]
Toivonen (2007) (Etoricoxib)	4/15	(26.7%)	0/15	(0.0%)	13.6%	9.0 [0.53, 153.8]
**Pooled Estimate**					I^2: 0%	3.0 [1.05, 8.54]

Mantel-Haenszel, DerSimonian-Laird Random Effects — *p=0.04, z=2.06, τ^2=0.00* — *RR: Risk Ratio, CI: Confidence Interval*

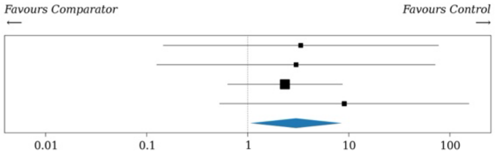

Figure A4. Forest plot (random effects) showing comparing rates of postoperative drowsiness between pre-emptive medications and placebo

Table A1. Search Criteria

**Search Criteria**
1. pre-medicate
2. preoperative
3. preventative
4. pre-incisional
5. pre-surgical
6. pre-emptive
7. 1 or 2 or 3 or 4 or 5 or 6
8. arthroscopy
9. arthroscopic
10. meniscectomy
11. meniscal
12. meniscus
13. Anterior cruciate ligament
14. Medial patellofemoral ligament
15. Rotator cuff
16. Shoulder instability

17. 8 or 9 or 10 or 11 or 12 or 13 or 14 or 15 or 16
18. analgesia
19. pain
20. analgesic
21. 18 or 19 or 20
22. 7 and 17 and 21

Table A2. Study designs and protocols of included studies.

Author (year)	Blinded (Y/N)	Placebo controlled (Y/N)	Postoperative Pain Protocol
1. Boonriong (2010)	Y	Y	Oral acetaminophen 1000 mg q6h PRN ± IV fentanyl 1 mcg/kg q3h as requested by patients.
2. Dahl (2012)	Y	Y	Oral acetaminophen 1000 mg QID starting ~6h post-op. Rescue IV fentanyl 50 mg for breakthrough pain in PACU. Codeine prescribed after discharge.
3. Degen (2023)	Y	Y	Standardized PACU regimen. Additional analgesia if VAS ≥ 4: IV fentanyl 25–50 µg (every 5 min up to a max 200 µg) and/or IV hydromorphone 0.2–0.4 mg PRN (max 4 mg). Once oral medications tolerated, PO oxycodone hydrochloride 5 mg/acetaminophen 325 mg PRN before discharge.
4. Ekman (2006)	Y	Y	PO celecoxib (200 mg) or placebo at first pain request. For optional additional pain medication, PO hydrocodone bitartrate 5 mg/APAP 500 mg 1-2 tabs q4-6h PRN.
5. Entezary (2014)	Y	Y	IV morphine q4h scheduled or PRN.
6. Hou (2019)	N	N	EPA group: PO meloxicam 15 mg 24h before surgery, 7.5 mg 1h before surgery, 7.5 mg at 24h post-op. POA group: PO meloxicam 15mg 4h post-op, 7.5mg 24h post-op. Rescue: IV pethidine PRN for intolerable pain from 24h pre-op to 48h post-op.
7. Kahlenberg (2017)	Y	Y	Standardized PACU protocol: PO acetaminophen-hydrocodone for mild/moderate pain ± IV hydromorphone for severe pain.
8. Kavak Akelma (2020)	Y	Y	Standardized PACU protocol: PO dexketoprofen trometamol 50 mg q12h + IV tramadol PCA (10 mg bolus, 10-min lockout). Patients with NRS score ≥ 4: IV tramadol 50 mg PRN.
9. Lierz (2012)	Y	Y	IV morphine PCA 0.5 mg/kg PRN in first 24 h, PCA setting (0.02 mg/kg/bolus dose, max 6 doses/hr).
10. Ma (2021)	N	N	PRE group: PO celecoxib 400 mg 2h pre-op, 200 mg 12h, 24h, 36 h post-op. PO meloxicam 15 mg 2h pre-op, 7.5 mg 24h post-op. PO rofecoxib 50 mg 2h pre-op, 25 mg 24h post-op. POST group: PO celecoxib 400 mg 4h post-op, 200 mg 12h, 24h, 36h post-op. PO meloxicam 15 mg 4h post-op, 7.5 mg 24h post-op. PO rofecoxib 50 mg 4h post-op, 25 mg 24h post-op. Rescue analgesia: IV/IM pethidine 0.5 mg/kg PRN.
11. Mardani-Kivi (2013)	Y	Y	IV/IM pethidine 0.5 mg/kg PRN in first 24h post-op.
12. Mardani-Kivi (2013)	Y	Y	IV/IM pethidine 0.5 mg/kg PRN in first 24h post-op.
13. Mardani-Kivi (2016)	Y	Y	IV/IM pethidine 0.5 mg/kg PRN in first 24h post-op.
14. Ménigaux (2005)	Y	Y	PO Ketoprofen 150 mg BID starting the evening of surgery. PACU: IV morphine 3mg q5min titration PRN until VAS ≤30 mm. IV morphine stopped if sedation score >2 or RR < 12 breaths/min. PCA morphine: 1mg bolus, 5-min lockout, no background infusion (x48h).
15. Montazeri (2007)	Y	Y	PCA IV morphine 0.05 mg/kg PRN in the first 24h post-op.
16. Nimmaanrat (2012)	N	Y	Post-op PCA morphine settings: IV morphine 1 mg bolus, 5-min lockout, 4h limit of 40mg, no continuous infusion.
17. Su (2022)	Y	Y	PO celecoxib 200 mg q12h starting POD1 x 2 weeks. If pain unbearable or VAS > 4: IM tramadol 100 mg PRN.
18. Toivonen (2007)	Y	Y	PACU: IV fentanyl 0.025 mg q5min PRN until VAS < 3. SDS: PO Panacod (acetaminophen 500 mg + codeine 30 mg), 1 tab PRN for VAS ≥ 3 (max 8/day).

19. Uribe (2018)	Y	Y	PACU rescue analgesic: IV hydromorphone 0.5mg PRN. Discharge prescription: PO ibuprofen 800 mg q6h PRN + PO oxycodone/acetaminophen 5/325 mg q4h PRN.
20. Zhang (2014)	Y	Y	PACU: “pain medications” PRN. PO hydrocodone 5 mg/acetaminophen 500 mg q4h PRN upon discharge.
21. Zhou (2017)	N	N	EPEA group: PO celecoxib 400 mg 24h pre-op, 200 mg 12h pre-op, 1h post-op, 13h post-op. PEA group: PO celecoxib 400 mg 1h pre-op, 200 mg 10h post-op, 22h post-op. POA group: PO celecoxib 400 mg 4h post-op, 200 mg 16h post-op. Rescue: IM/IV pethidine 5 mg/kg PRN during entire 36h observation.
22. Zhu (2020)	N	N	Preemptive group: PO celecoxib 400 mg 24h pre-op, 200 mg 12 h pre-op, 2h pre-op, 200 mg BID POD1-7. Postop group: PO celecoxib 400 mg 12h postop, then200 mg BID POD1-7. Rescue: IV/IM pethidine PRN for intolerable pain. Prophylaxis: PO celexocib 200 mg QD POD8 to 1 month.
